# Exposure to H1 genotype measles virus at an international airport in Japan on 31 July 2016 results in a measles outbreak

**DOI:** 10.5365/wpsar.2016.7.4.007

**Published:** 2017-02-07

**Authors:** Aika Watanabe, Yusuke Kobayashi, Tomoe Shimada, Yuichiro Yahata, Ayako Kobayashi, Mizue Kanai, Yushi Hachisu, Munehisa Fukusumi, Hajime Kamiya, Takuri Takahashi, Yuzo Arima, Hitomi Kinoshita, Kazuhiko Kanou, Takehito Saitoh, Satoru Arai, Hiroshi Satoh, Hideo Okuno, Saeko Morino, Tamano Matsui, Tomimasa Sunagawa, Keiko Tanaka-Taya, Makoto Takeda, Katsuhiro Komase, Kazunori Oishi

**Affiliations:** a Field Epidemiology Training Program, National Institute of Infectious Diseases.; b Department of Epidemiology for Infectious Diseases, Graduate School of Medicine, Osaka University, Japan.; c Infectious Disease Surveillance Center, National Institute of Infectious Diseases.; d Department of Virology III, National Institute of Infectious Diseases.; e Division of Global Infectious Diseases, Department of Infection and Epidemiology, Graduate School of Medicine, Tohoku University, Miyagi, Japan.

## Introduction

In March 2015, the Measles Regional Verification Commission for the World Health Organization Western Pacific Region verified that Japan had achieved measles elimination (*1*) based on the verification criteria. (*2*) Only 35 confirmed measles cases were reported in 2015, and for 2016, measles activity was low until July (*n* = 16, as of 3 August). However, the number of reported measles cases surged in the middle of August 2016. Several cases were considered sporadic cases without a known source of infection or imported cases because they initially seemed to be unrelated. However, through vigilant daily monitoring of national surveillance data by surveillance officers at the national level, including fellows of the Field Epidemiology Training Program at the National Institute of Infectious Diseases, and their close communication with local public health staff, five cases were found to have been present at a large international airport on the same day as a possible index case was found.

### Recent measles situation in Japan

Measles became a case-based notifiable disease in 2008. The case definition for measles used in national surveillance is based on clinical symptoms and laboratory tests. The diagnosis of measles is confirmed by laboratory test results, including a positive result for measles-specific immunoglobulin M (IgM) titre, significant increase in measles-specific immunoglobulin G (IgG) titre using paired serum, the detection of measles virus (MV) by reverse transcription polymerase chain reaction (RT–PCR) or isolation of MV in cell culture. MV detection, isolation and genotyping are performed mainly at designated local governmental (i.e. municipal or prefectural) public health institutions within each local government area. The number of reported measles cases in Japan has declined markedly from 11 013 in 2008 to 35 in 2015. (*3*) The D5 genotype strain of MV, which was endemic in Japan, has not been detected since May 2010; however, limited local transmission following importation of MV has been observed, as in 2014. (*4*)

### Common exposure to H1 genotype MV at an international airport

In 2016, although measles activity remained at the lowest level since 2008, the number of reported measles cases surged in epidemiological week 33. (*5*) Surveillance officers and Field Epidemiology Training Program fellows noted five measles cases (**Table 1**, cases 1–5) with close onset dates reported from different prefectures that seemingly did not have any common exposure history. Case 1 was a ground crew member at Kansai International Airport (KIX) in Osaka Prefecture, the third largest international airport in Japan, handling about 64 000 passengers per day. (*6*) This patient had no recent history of overseas travel. Case 5 had travelled domestically before the onset of measles. The other three (cases 2–4) had travelled to Indonesia, the Republic of Korea and Viet Nam; they were initially suspected to have become infected with MV at their destination. However, MV was confirmed in all cases by RT–PCR and determined to be the H1 genotype strain, the predominant genotype reported from China and parts of South-Eastern Asia over the past three years. (*7*)

**Table 1:** Cases with H1 genotype measles virus likely acquired at Kansai International Airport, July–August 2016

Case	Age group	Sex	Vaccination history	Travel history (destination and period)	Onset date (fever or rash)	Reporting prefecture	Reported date at KIX*
1	20–24 years	F	Unknown	None	9 Aug	A	31 Jul
2	15–19 years	M	None	Indonesia, 31 Jul–5 Aug	9 Aug	B	31 Jul
3	25–29 years	M	Unknown	Viet Nam, 31 Jul–6 Aug	10 Aug	C	31 Jul
4	30–34 years	M	None	Republic of Korea, 31 Jul–2 Aug	10 Aug	D	31 Jul
5	40–49 years	M	None	Domestic travel, 31 Jul–3 Aug	10 Aug	E	31 Jul

* KIX = Kansai International Airport

Because the genotype H1 strain is not endemic in Indonesia, Japan or the Republic of Korea, we obtained epidemiological information from local health authorities at each reporting prefecture to clarify travel itineraries, including domestic transit, of the five cases. We found that these five cases had spent time on the same floor of KIX on 31 July 2016. Sequence analysis revealed high nucleotide sequence homology between the H1 genotype MV strains detected in the five cases. Based on these findings, we concluded that KIX was the likely place of exposure.

### Alert to the general public

The National Institute of Infectious Diseases (NIID) and the Ministry of Health, Labour and Welfare of Japan announced an increase in the number of measles cases in late August 2016 to remind the general public to get vaccinated and to raise physicians’ awareness (i.e. to consider measles when examining patients with fever, rash, and travel history and/or epidemiological information such as contact with people displaying measles-like symptoms during the incubation period). In addition, information about the cases suspected to have been exposed to H1 genotype MV on 31 July 2016 at KIX were posted on NIID’s web site to inform the general public and health-care providers of the risk of exposure to MV at KIX.

### Possible source of the H1 genotype MV at KIX

In late August, person A (sex not disclosed) reported information that provided insight into the source of exposure at KIX. MV infection was confirmed in person A by measles-specific IgM. Person A reported having contact before measles onset with person B, who had returned from China to Japan on 20 July 2016 and developed measles-like symptoms on 26 July. Person B (sex not disclosed), who had visited KIX on 31 July, consulted physicians and was diagnosed with the common cold and/or drug eruption before measles-specific IgM was confirmed. Given that person B returned from China during the measles incubation period and visited KIX while symptomatic on 31 July, this person was considered to be the possible source of MV for all five cases, even though the confirmation of genotype H1 strain was not obtained from the case.

### Additional cases due to transmission at KIX

Following further investigations, the Osaka Prefecture local government reported on 31 August an additional 16 laboratory-confirmed cases, all of whom shared a single office at KIX with case 1 (Table 1), a ground crew member. The outbreak investigation in this office was conducted and its findings will be reported elsewhere.

## Discussion

This cluster reminds us that an international airport is a potential hotspot for measles and may act as a mixing place for travellers from measles-endemic countries and any unvaccinated non-immune persons, as reported previously. (*8*-*10*) As of 7 December 2016, (*5*) no additional cases related to this KIX cluster have been reported, and the numbers of both suspected and confirmed cases have been declining. However, authorities should remain vigilant about the risk of importation of MV from endemic countries. High-quality surveillance and high vaccination coverage must be continued for Japan to preserve measles elimination status.
